# Gender Selection Dilemma in Fast Moving Consumer Goods (FMCG) Advertising: Insights from Eye- Tracking Research

**DOI:** 10.16910/jemr.17.2.6

**Published:** 2024-07-22

**Authors:** Minanshu Sinha, Madhvendra Misra, Saurabh Mishra

**Affiliations:** Department of Management Studies, Indian Institute of Information Technology Allahabad, India

**Keywords:** Eye-Tracking, Celebrity Endorsement, FMCG, Neuromarketing Webcam Based Eye-Tracking, Spokesperson Gender

## Abstract

Selecting the gender of a celebrity for fast-moving consumer goods (FMCG) advertising presents a strategic challenge. Previous research has predominantly concentrated on comparing celebrity spokespersons with non-celebrities, frequently neglecting the intricate distinctions in the effectiveness of male versus female endorsers. This study addresses this research gap by employing both traditional and neuromarketing methodologies. By integrating eye-tracking technology via RealEye and questionnaire-based surveys, the results indicate that female celebrities are more effective in capturing visual attention, whereas male celebrities are more effective in enhancing perceived trustworthiness. These findings are pivotal for both academic research and commercial strategy, as they elucidate the optimal selection of celebrity gender for maximizing FMCG advertising efficacy.

## Introduction

Businesses employ cutting-edge strategies to survive the intense competition and maintain a sizable market share (42). It is done by using marketing communications to draw in customers (61). Advertisement is the key part of the promotion component of any brand (83),because it plays a vital role in influencing the choice making (7). In the growing business world, the marketing practices are getting thinned by global competition in almost every sector (25). People around the globe are essentially associated with one sector which is fast-moving consumer goods (FMCG; further referred to as these are the products of low involvement, low price, frequent purchase, short shelf life, sold in supermarkets and hypermarkets) during everyday life (73). After all, the majority of these products deal with every day, low-involvement consumer decision-making (3).

Advertisement and its effectiveness in the FMCG sector have become the most challenging due to the large pool of choices (76). Consumers frequently choose brands for FMCG without conducting extensive research or comparisons. The big challenge that FMCG brands faces are the reach and retention of the consumers (83). Majority of the FMCG advertisements are endorsed by celebrities. When looking for FMCG advertising efficacy, the choice of a celebrity spokesperson depends on the primary specified goal of marketing communication. In every media, celebrity endorsement has increased drastically(39). According to the available studies, celebrity endorsement largely reinforces the consumer's propensity to buy (4; 34; 74). Since celebrities have a distinct fan base, businesses aim to use their notoriety to draw in a broad and varied customer base. It is increasingly typical to see corporations engage celebrities to promote their brands to persuade people to buy their items. For example, Stacy Jones, founder and CEO of Hollywood Branded, a marketing firm that promotes "pop culture partnerships," notes in a study carried out in the United States of America that customers have a history of embracing celebrity trends and using the things that people they admire and aspire to use (65). A celebrity promoting your company gives you a presence that can have an effect on your sales and raise consumer awareness of your brand (92). For that reason, there has been an increase in the number of academic studies looking into celebrity endorsements. Celebrity endorsement helps to connect with the target demography and quickly enhances audience attention (56)

In India, celebrities are involved in about 50% of endorsements in advertisements with brands and products (Techsci Research, 2023). According to a report from TAM AdEx 2022, the number of television commercials starring celebrities climbed by 60% between 2020 and 2022 (66). By 2025, it is anticipated that rising internet usage in India will further fuel the market for celebrity endorsements. The market for celebrity endorsements in India is anticipated to expand at a strong rate during the forecast period as a result of changing consumer lifestyles and the expanding power of media and communication (17). Additionally, the growing use of social media combined with the growing urban population has made these digital platforms valuable marketing tools (48).

A celebrity endorser, according to Choi and Rifon, is "any person who enjoys public recognition and who uses this recognition on behalf of a consumer good by appearing with it in an advertisement" (13). It provided a deeper understanding of celebrity endorsement's impact on consumers' purchasing decisions (53). Some endorsements are successful at persuading customers to purchase the product or support the cause they are endorsing (9). Studying celebrity endorsement, it is found that the two main components of a source credibility, perceived trustworthiness and competence (32) and celebrity attractiveness from source attractiveness theory constitutes the main variables. The definition of source credibility is, the amalgamation of knowledgeable and reliability in a particular field that is regarded as trustworthy (14; 85). A source is rated based on three criteria: expertise, physical beauty, and trustworthiness, following the SCM (62).

A celebrity commercial may draw more attention, which could deepen the processing of the product–celebrity pairing and make it easier to recall specific experiences connected to the celebrity. Attention may be crucial in celebrity endorsement scenarios since the recollection of explicit memories may be necessary to demonstrate a positive affect transfer from celebrity to goods (81). According to Febriyantoro, attention promotes the growth of brand awareness, which shapes attitude and influences purchase intentions (23). Despite the abundance of literature on the subject, there are divergent opinions regarding the characteristics of spokespersons that increase the efficiency of advertising for various product categories; often, these opposing viewpoints center on celebrity and non-celebrity spokespersons. Studies conducted in celebrity endorsement are majorly focused on the learning and effectiveness differences between the celebrity and non-celebrity endorsed advertisements in the field of tourism, destination advertising, B2B advertising, FMCG. For instance, a study conducted by revealed that the attentional resources required to process advertisements are higher for celebrity spokespersons than for non-celebrity spokespersons. Also, Ferguson and Mohan found in their study on B2B employees that although managers are more likely to notice an advertisement when a celebrity endorser is included, managers who spend more time focused on the advertisement are also more likely to have unfavourable hedonic sentiments toward it (24).

A famous sponsor can also lessen the negative utilitarian perceptions of the advertisement. With a non-celebrity in the advertisement, brand recall was highest. Stallen et al. exposed young female individuals to images of products along with equally beautiful pictures of celebrity and non-celebrity faces using a functional magnetic resonance imaging (fMRI) scanner, which recorded their brain activity (81. They found out that female celebrities were more attractive than non-celebrities as perceived. In a similar manner, some studies were also between local celebrities and source celebrity, local celebrity, and host celebrity. Li et. al. conducted a pretest and three experiments using a source celebrity and a local celebrity involving eye-tracking results and lab experiments and established that using a source celebrity in international destination marketing (as opposed to a local celebrity) can greatly enhance traveler’s positive attitudes toward the destination, intentions to visit, and visual attention to the advertised scenery (51). Additionally, the study done in Korea by B. Liu et al. provides a deeper understanding of the efficacy, underlying mechanisms, and boundary conditions of various forms of celebrity endorsement in the context of marketing tourism destination (52). It is based on the match-up hypothesis and uses a mixed methods approach that combines eye tracking technology and self-report techniques. The study has given empirical proof that utilizing an origin celebrity instead of a host celebrity in an endorsement advertising campaign can successfully raise emotional arousal, which in turn raises travel intention.

This article focuses solely on the gender of celebrity endorser, due to the lack of scientific research on the topic of using female versus male celebrity spokespersons as FMCG spokespersons. It also tackles the issue of spokesperson selection using neuromarketing and traditional marketing together in order to help the marketer decide as celebrities charge a very large sum of money and to know whether their money would result in higher effectiveness or not is very essential. The reason for using traditional method is that some scientists say that measuring attention is not just enough to study to deduce a conclusion (18; 31). Firstly, purchase intention and celebrity credibility by questionnaire method would be measured. Neuromarketing would help the marketing to understand the attention pattern of the consumer. Therefore, we are using eye tracking, more especially, to look into the issue. Eye tracking has been used to solve many problems in past like pun processing in advertising posters (46), task complexity influence gaze patterns during landings using visual flight rules among inexperienced pilots (5), depression detection (84), pianists’ eye-hand span and visual integration (10), virtual reality (69), music industry (67), brain injury (59), academic performances (75), etc. Here a web-cam based eye tracking has been used which is a software that runs on web browsers and data is collected. Fixation count and dwell time are measures to know the attention of the consumer on celebrity. Fixation count will measure the total number of times a respondent looks on a certain area of interest (AOI). Dwell time the total time taken by the respondents to see the AOI which includes fixation duration and saccade movement.

There are mainly three objectives of the study. First one is to measure the attention on celebrity endorsement of FMCG products. The second one is to measure the credibility of the endorser of the FMCG product in the print advertisement. The last one is measuring the purchase intention of the FMCG celebrity endorsed product. The first objective is fulfilled by using state of the art eye tracking measures which quantifies the amount of attention given to the area of interest (reference). The next two objectives are achieved by using a self-reported questionnaire. This study answers three questions, a) Does gender have an effect on purchase intention, if yes then which one is more effective; b) Which gender is more trustworthy; c) Which gender is attractive and lastly, which gender has a higher level of expertise. Thus, by answering and evaluating these factors we would be able to decide which gender of the celebrity would be best to cast. This would contribute to the effectiveness of the advertising thereby saving money.

This study would increment the academic literature by studying between the male and female celebrity endorsement using eye tracking and provide recommendations for the selection of a male or female advertising spokesperson as it is very difficult as well as important for the marketers to choose between the two genders in different stages of product lifecycle.

## Literature Review

FMCG products are highly sought-after and most commonly purchased in India, owing to their continually strong demand. This competitive market consists of several renowned Indian and foreign FMCG brands competing for a portion of the market. Consequently, advertising for fast-moving consumer goods (FMCG) in India is crucial to upholding a prominent position in the minds of consumers. Throughout the year, one can see a diverse range of major FMCG advertising campaigns across many platforms, such as television, digital media, newspapers, radio, outdoor billboards, cinemas, and magazines. As of Tanushree Basuroy’s report from Statista 2024, television and digital media each represented 47 percent of the total advertising expenditure in the fast-moving consumer goods (FMCG) sector across India (6). Given that India is a developing market with a population of 1.4 billion people, as reported by the World Bank and the US Census Bureau, about 210 million Indian households currently own a television, around 6.9% from 197 million in 2018, according to the most recent estimates from TV monitoring group BARC (Broadcast Audience Research Council) India. According to Nielsen’s India Internet Report 2023, there were 425 million internet users in rural India, over 44% more than there were in urban areas (295 million users).

Advertising and marketing firm Dentsu predicted in a report that the Indian advertising business would grow by 14.7% to $12.6 billion in 2023. Dentsu India's 2023 study states that the FMCG industry accounts for 30% of all advertising expenditures, with internet retail coming in second at 18%. A crucial component of marketing strategies for companies looking to raise consumer awareness of their products in India is celebrity endorsements. Here, celebrity endorsements have been prevalent for a very long time, and it is becoming harder for firms to hold consumers' attention.

According to Dodrajika, to draw attention to the message, advertisers frequently use famous people who are well-known to, frequently liked by, or even idolized by, their target audience (21). Examples of these people include actors and actresses, sports, entertainers, and other well-known public personalities. The academic literature in advertising is replete with evidence of the beneficial impacts of incorporating celebrities on both ad and brand assessments (62), the majority of which uses the time-tested "source credibility models" (22). Customers and the famous people who endorse the items have a Para social relationship, also known as the "illusion of intimacy" (55). However, not all celebrities are suitable for brand promotion (44). It is fascinating to understand the mechanisms behind endorsements given the wildly different consequences of celebrity endorsements as a result of the engagement of a variety of elements, including celebrity choosing, advertisement quality, media planning, and press relations. The celebrity's gender may also be critically important to the success and appraisal of the brand (38).

One of the key metrics for measuring the success of advertising is the capacity to capture consumers' attention (47). Thus, the spokesperson serves as a major element of overall advertising efficacy by grabbing customers' attention (40). A crucial responsibility is selecting an appropriate spokesperson. While an ineffective spokesperson may have little to no effect or even detrimental effects, an effective spokesperson can boost sales, improve consumer perception of a product, and build brand value. As a result, the impact of various spokespersons will vary depending on the market and the product. Attention based marketing (ABM) permits to directly measure visual attention to advertising stimuli by using eye-tracking technology (51). According to Rayner et al., studies have demonstrated that facial photos in ads draw attention more quickly than other types of stimulation (70). According to Todorov et al., individuals make judgements about a person's personality based on their face features (86). Additionally, Sigurdsson H emphasize that a person's face familiarity may be taken into account when determining whether or not to like and trust them (80). Consumers first encounter a spokesperson's face within the context of advertising (54). As a result, one of the key markers of successful advertising could be the selection of the spokesperson (71). Additionally, the spokesperson for the advertisement can have a big impact in grabbing and holding viewers' attention (88).

While some research have indicated that the gender of the product endorser has a substantial impact on consumers' purchase intention towards the products, others dispute it (8). Previous research on the gender of the main character has produced contradictory findings. There appears to be little difference between studies that reported a preponderance of female primary characters and those that showed a majority of male primary characters (28). For example, a study conducted by Del Saz-Rubio examines the portrayal of male identities in television advertisements for male toiletries through a pragmatic and multimodal analysis. The study conducted empirical testing on implicit assumptions using a sample of ten male informants. These assumptions were then grouped into thematic cores for subsequent analysis. The findings indicate that the advertisements depend on stereotyped and conventional notions of masculinity, promoting the use of grooming goods in a manner that is perceived as traditionally masculine. These advertising emphasize attributes like as sexual prowess, physical strength, toughness, and resourcefulness. These depictions present a limited perspective on present-day males, disregarding the various roles they fulfill in today's society (20). Nonetheless, the majority of research suggests that male protagonists are more common. Whereas, another study by Paprina reveals gender differences in advertising responses for low-involvement products. Females process ads more detailed, while males heuristically. This affects word-of-mouth communication, with cognitive responses mediating the effect (64). Also, the study carried out by Scanlon in 2013 analyzes the endeavors of women at J. Walter Thompson during the early 20th century, with a specific emphasis on their contributions to the field of advertising. The J. Walter Thompson Women's Editorial Department played a crucial role in advancing women's prospects in advertising, an industry that was predominantly male-dominated during the early 20th century (77). Also, studies in the context of the stock market, in particular, demonstrate that the gender of the celebrity endorser has little bearing on the financial results of the endorsed company (90). While some studies on celebrity chef endorsement, confirm that consumers prefer male celebrity chefs over female celebrity chefs in terms of the effectiveness of their advertising (36), other studies in various contexts confirm that consumers find female celebrity chefs to be more credible than male celebrity chefs (16). Consumers prefer to purchase GPT brochures with photographs of female tour leaders rather than those with photos of male tour leaders (68). And the effectiveness of FMCG advertising is significantly impacted by well-known female spokespersons (11).

In light of the foregoing debate, it is therefore proposed that:

H1: There exists a statistically significant gender-based disparity in the allocation of visual attention by consumers, with a greater proportion directed towards advertisements featuring female celebrities than male celebrities.

Customers who focus more on a spokesperson in an advertisement may have stronger purchasing intentions. On the scale of attractiveness both the celebrity and the non-celebrity females were perceived as being more attractive females than average. And celebrities’ likeability ratings were more than half. And there was no significant difference between the purchase intention of the female celebrity and non-celebrity. The FMRI study conducted in 2010 by Mirre Stallen et. al. on the shoes endorsed by female celebrity revealed that increased activity in the medial orbitofrontal cortex, which is consistent with the idea that pleasant emotions are triggered by celebrities (81). The medial orbitofrontal cortex is also linked to the encoding of the subject's preference for stimuli (72).

H2: The level of purchase intention generated by advertisements featuring male celebrities is statistically higher than that resulting from advertisements featuring female celebrities.

The ideas of source credibility (35) and source attractiveness (60) serve as the main theoretical foundations for understanding communication efficacy. Communicator credibility is defined by source credibility theory as the extent to which a "source is perceived as possessing expertise relevant to the communication topic and can be trusted to give an objective opinion on the subject" (29). This definition emphasizes knowledge and dependability as crucial components of source credibility. While expertise denotes a person's aptitude and competency in delivering true and precise information, trustworthiness shows a source's perceived honesty and dependability. According to McGuire's (60) source attractiveness theory, attractiveness is a third attribute of a reliable communication source.

Also, according to AlFarraj et al., perceived trustworthiness, expertise and attractiveness are the celebrity credibility dimensions (2). Wherein, perceived trustworthiness refers to impressions of an endorser's honesty, integrity, and believability, whereas expertise refers to the pertinent information, skills, or experience the endorser is thought to possess (79). In contrast to expertise and trustworthiness, which both pertain to the character and are usually used to allude to "credibility," attractiveness primarily refers to how one judge's physical appearance (14).

Accordingly, studies have shown that female spokespersons are more effective for appealing to female clients (43), while male celebrities are more appealing to male ones (45). The opposite is also true: research has demonstrated that gender incongruity will result in improved consumer behavior results (57). These contradictory results have raised the question of whether practitioners favor female celebrity endorsing over male celebrity endorsing. Hence, it is hypothesized

H3:

a) Male celebrity endorsements in advertising exhibit statistically higher perceived trustworthiness ratings compared to female celebrity endorsements.

b) Male celebrities garner statistically greater perception of attractiveness ratings in advertisements than females, contributing to their overall credibility.

c) Ads featuring male celebrities statistically demonstrate greater perceptions of expertise in comparison to ads with female celebrities, enhancing product credibility.

A consumer's attention can signal the start of their purchasing behavior and influence their subsequent evaluation, judgement, and product selection during the shopping process. An advertisement's ability to grab viewers' visual attention is a key sign of its efficacy (51).

H4:

a) Purchase intention is positively associated with the level of attention given to male celebrities.

b) The greater the focus on a female celebrity, the stronger the inclination towards purchasing.

Research on visual attention and advertising effectiveness has revealed various insights into how different ad elements and formats influence consumer responses. Zhang and Yuan (93) monitored three key components of advertisements—product, brand, and endorser—using eye movement indicators such as transformed fixation time (TFT), transformed fixation number (TFN), and average gaze duration (AGD) (93). Their study found that eye movements positively correlated with the effectiveness of product and endorser elements but negatively with brand elements . Similarly, a study by De Keyzer et al. (19) employing eye-tracking technology with 90 participants demonstrated that native advertisements attract greater and more sustained visual attention than banner ads (19). This heightened attention, measured by total fixation duration and average visit duration, enhances the understanding of persuasive techniques and ad recognition, thereby boosting brand recognition. Additionally, Lee and Ahn (49) investigated the impact of visual attention on Internet banner ad efficacy, focusing on how different attention levels affect conscious and unconscious reactions (49). Their findings indicate that while animation in ads does not necessarily improve attention, it can still influence user attitudes toward the brand unconsciously, even if the ad is not consciously noticed. Together, these studies highlight the complex interplay between visual attention, ad elements, and advertising effectiveness. Moreover, advertisements showcasing celebrities have proven effective in capturing consumers' attention and conveying messages swiftly and with assurance (47). In the same context attention is supposed to be positively associated with celebrity credibility. Hence, we hypothesize that,

H5: The level of attention directed towards male celebrities is positively linked to their credibility as public figures.

a) Perceived Attractiveness

b) Perceived Expertise

c) Perceived Trustworthiness

H6: The prominence of attention given to female celebrities is positively associated with their credibility in the public eye

a) Perceived Attractiveness

b) Perceived Expertise

c) Perceived Trustworthiness

It is more probable for consumers to base their decisions on subconscious thinking. Thus, eye-tracking and other neuroscience technologies have been used (51). The easiest technique to assess attention essentially is with eye-tracking equipment (27). It was determined by two metrices, fixation count and dwell time (87). Fixation count is the count of how many times viewers watched various target spokespersons and products placed in various positions (which made up the areas of interest). And the whole time spent looking within an AOI is called the dwell time (30). This covers every fixation and saccade—including revisits—that occurs during the AOI. Dwell time and fixation count is a great indicator of how interested someone is in a certain AOI (91). It seems to reason that interest in the AOI will increase with dwell duration (27).

RealEye is online software that records gaze positions using a standard webcam and a web browser. For facial landmark and gaze detection, the eye tracker makes use of the client machine. A JavaScript-based eye tracking engine is executed by the web browser. TensorFlow.js and a face landmark model were used to enhance and customize the WebGazer foundational software, which is distributed under the Apache License 2.0 (63). Webcam access uses the JavaScript Media Devices API to set resolution to 1440x900 at a minimum frame rate of 30 frames per second and up to 60 frames per second if the webcam supports it. RealEye uses a fixation filter method resembling the I-VT (Velocity-Threshold Identification) filter, averaging data at a sampling rate of over 20 Hz, with a minimum fixation duration set by default to 100 ms. For noise reduction, a median filter is employed, with the default value set at 21.

In the next section, the use of neuromarketing and conventional marketing research techniques to test the hypothesis will be discussed.

## Methodology

The study is divided into two parts, first one is the eye-tracking study and the other one is the traditional method which is self-reported measure in a laboratory setting simulation of real world (26). The experiment was conducted in Indian Institute of Information Technology, Allahabad, India and the participants recruited were the students of from the same institute. But before that, respondents were asked to identify their age and gender, spoken language (50), Geographical Location (Place you belong to), Nationality, Education (Completed Degree) and Eye Vision, severe visual impairments, neurological or psychiatric disorders, current drug use, distress at being still (Table 2).

**Figure 1.**
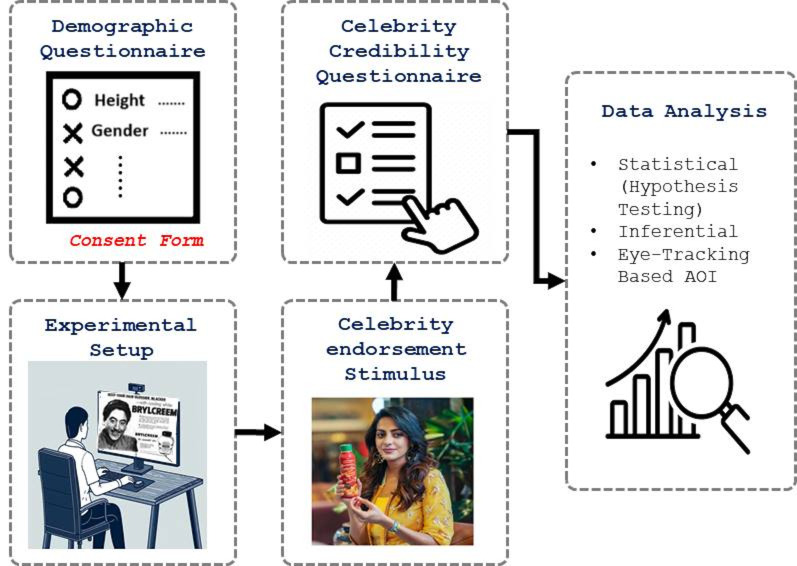
Flowchart of the Experiment

Each participant was instructed with the procedure of the experiment and consent was taken from everyone prior to the experiment. For taking part in the eye tracking research, each volunteer participant received 10 Rupees. Prior to the experiments, each participant received thorough information about the study and completed the informed consent form. Four target stimuli, a distractor stimulus, and a standard stimulus made up the visual stimuli.

The instructions given to the participants was that they will participate in an eye tracking experiment where they view four visual stimuli and complete a questionnaire. The process begins with an introduction and consent form, followed by a comfortably sitting in front of a computer monitor with an eye tracking device which is web-cam in this case. After this calibration would start. Calibration is crucial for accurate tracking data, and participants must focus on specific points on the screen. After calibration, participants are shown four stimuli one at a time, with a brief break between each. A questionnaire will be provided, asking participants about their impressions and experiences. Post-experiment, participants are encouraged to provide honest feedback and contribute to the research.

**Table 1:**
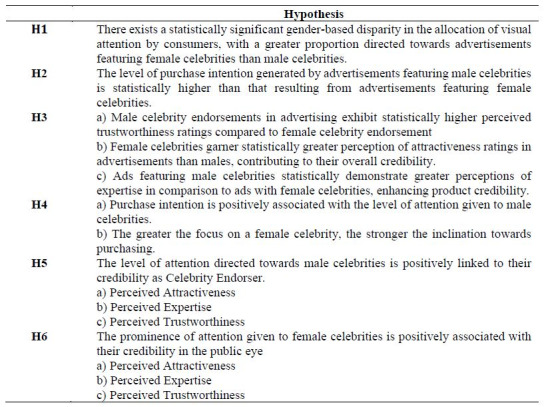
Hypothesis Testing Table

### Stimulus

The first target stimulus featured a well-known male celebrity Bollywood actor in India with a known brand of energy drink; the second target stimulus featured a male celebrity who is an Indian cricket player with a different known brand of snacks. The third is a female celebrity is a Bollywood actress endorser with a known toothpaste brand. The fourth was a known soft drink brand with a female celebrity who is a famous Tennis player. The advertisements were taken from Google Images and these photos were exempted from copyright according to The Copyright Act 1957, India.

 The mechanism of selecting the experimental stimuli, first criteria was to select the two male and two female celebrities so as to balance the gender ratio of the endorser. Second was that the celebrities must be holding the FMCG Product. Third was that, they need be endorsers of India. Fourth criteria were, since the literature and reports from TAM AdX suggest that actors from Indian Cinema are the most casted endorser and the second category was sports. Therefore, two celebrities who are actors of Indian cinema each male and female were chosen and particularly the two been taken as stimuli are most followed actor and actress on social media collectively has been selected. In the same way sports celebrities have been selected for the stimuli. Fifth criteria was that celebrity had to be a well-known Indian celebrity, so we chose them on the basis of the maximum number of followers on Facebook, Instagram, and Twitter collectively. After selecting the celebrities, the next task was to search print ads endorsed by the with FMCG products and products did not have to be gender specific as to avoid biasness. Sixth criteria was that the prints advertisements need to be real advertisements so as to observe the actual responses of the participants. Numerous real digital print advertisements were found and four of them were the most appropriate according to the study’s criteria.

**Figure 2.**
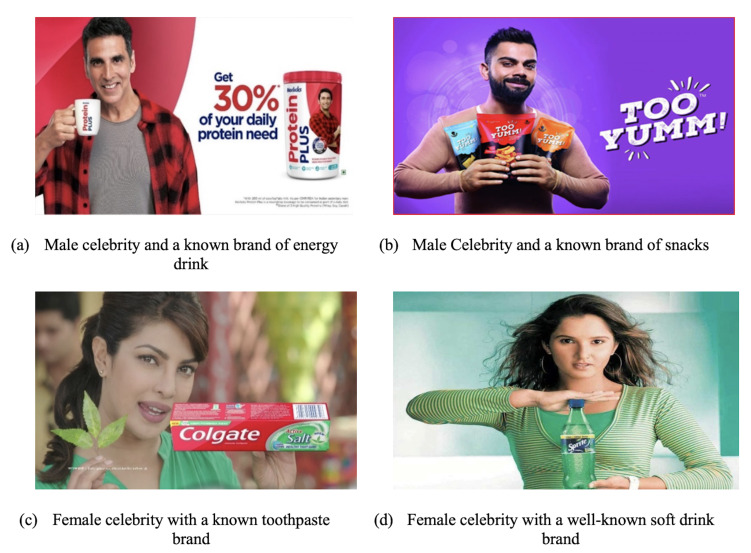
Celebrity images shown to the subjects for the analysis of FMCG consumer behavior

### Participants

Participants in an eye tracking study must be between 18 and 27 years old, have normal vision, fluency in language, basic computer knowledge, and be in good general health. They must be informed consent and follow experimental protocols. Exclusion criteria include severe visual impairments, neurological or psychiatric disorders, eye movement problems, current drug use, distress at being still, language differences, and previous involvement in similar experiments within the last six months. Participants should be fluent in the language used for the stimuli and questionnaire, have good general health, and not have any neurological or psychiatric disorders that could impair eye movement or visual perception.

### Eye-Tracking

Eye tracking is widely used in different disciplines like neuromarketing, neurolinguistics (58), etc. There are many types of eye-tracker, infrared eye-tracker, mobile eye-tracker (78), glasses eye tracker and web-cam based eye tracker. In this study, a web cam-based eye tracking, RealEye has been used which is the less expensive and portable alternative of the infrared eye tracking method which does not require much of a set up and high-end hardware. Neither does it require highly skilled professionals to execute the experiment. Merely one hour of training helped to understand the calibrations process and the data collection. We measured the fixation count and dwell time, one of the most measured constructs to measure attention. And the other constructs are obtained from the questionnaire method. To collect the data, consent was taken from the participants and data was collected using eye tracking and questionnaire. Data analysis was done by using in-built RealEye software , XLSTAT 2014 and SPSS v 25.

**Figure 3.**
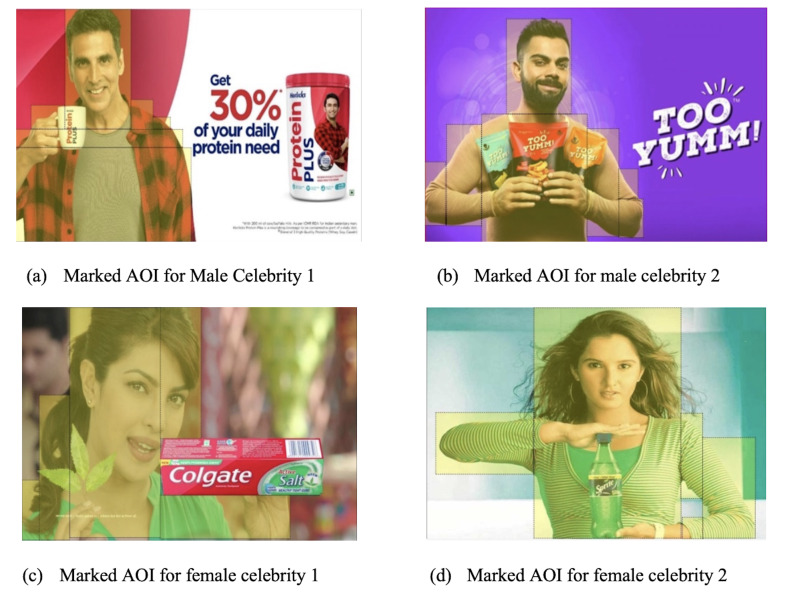
Defined Area of Interest (AOI) over the images of the celebrity endorsers

RealEye is used to set up the experimental method and to gather data in the webcam recording condition. To collect data that was most accurate for the normal recording environment, we decreased the sampling rate to 30 Hz. Eye movements were captured using a 1080P 2 Megapixel CMOS camera in Lenovo 300 FHD webcam. The eye tracing results were in the form of csv files analyzed using RealEye software. An online platform is provided by RealEye software for the planning and execution of the study. It helps online data analysis by estimating real-time gaze/fixation on Areas of Interest (AOIs).

Every recruited participant was made to sit comfortably in front of the computer screen which was 40cm away (12) of resolution 1440x990 and asked to fill in the initial questionnaire. After that, nine-point calibration was done by each participant as shown in figure 1. Calibration is important because every individual is different and different in eye measures as mentioned in the literature (82). The calibration was carried out in three backgrounds, black, white, and grey to avoid biasedness in the result due to the background light. Every advertisement stimulus was marked with the Area of Interest (AOI) to study the fixation count as shown in the figures. These AOIs were invisible to the participant. Using the RealEye software tool, the participants were shown the stimuli in a generated order. Every stimulus was shown for 10000 ms at fixation, in a controlled environment with room-temperature and normal lighting for a total of 698 trials. After watching the advertisement, a series of questions based on the ads were then posed as shown in figure 1 which is covered next.

The data from RealEye were statistically analyzed using the XLSTAT software package.

### Self-Reported Study

*Questionnaire*

An inclusive and exclusive question on the survey left out some of the recruited respondents. The questionnaire was completed by 100 qualifying respondents in total. After preliminary evaluation of the questionnaires that have been completed, replies with missing values of at least three were deleted in accordance with Hartline et. al. and Karikari et. al., resulting in a valid survey response rate of 86 percent and 86 useable surveys (33; 41). Out of which 86 results have been taken into consideration out of which 43 were of male and female each. A questionnaire study was done to examine the variations between attention, celebrity credibility as well as buy intentions about the brand promoted by various spokespersons as shown in figure 1. The questionnaire was divided into two main sections:

i) *Celebrity Credibility*

This section contained three parts and every part consisted of five questions concerning perceived trustworthiness, attractiveness, and expertise:

On a five-point Likert scale of from strongly disagree to strongly agree from 1 being the lowest and 5 being the highest. Perceived trustworthiness had five parts namely honest, dependable, reliable, sincere, and trustworthy. In the similar manner perceived attractiveness had five parts which consisted of beautiful, classy, attractive, elegant, and appealing. And the last is expertise which again consisted of five parts namely, expert, experienced, knowledge, qualified and skilled.

For example,

The celebrity that appears in the advertisement is Classy.

The celebrity that appears in the advertisement is dependable.

The celebrity that appears in the advertisement is expert.

ii) *Purchase intentions*

The strength and direction of a person’s buying intention towards the product were revealed by measuring the attitudes on a semantic differential scale. The intention was measured by four items each:

Product – probable, likely, certain, and chance,

How likely are you to purchase the product?

How probable is that you would purchase the product?

How certain are that you will purchase the product?

What is the chance that you will buy the product

In this way questionnaire survey has been taken up along with the eye-tracking. The next section contains the results obtained from the study.

## Results

### Socio-demographic Data

Using the software packages XLSTAT 2014 and SPSS V 25, descriptive and inferential statistical analyses were carried out to evaluate the variations in attention, celebrity credibility, and purchase intentions regarding the products advertised by male and female celebrity spokespersons. Data from 86 participants (43 male and 43 female) in eye tracking trials were also used in the analysis. The participants had uncorrected or normal vision. Out of the 86 participants, seventy- seven were between the ages of 18 and 25, and 9 were over the age of 25 (Table 2).

**Table 2:**
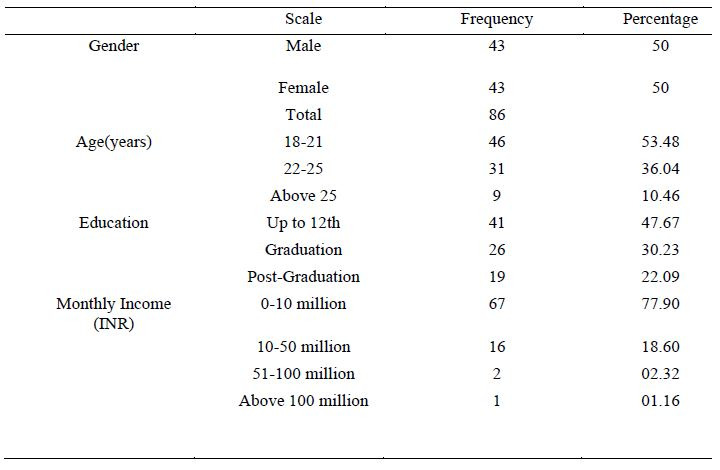
Demographic Data

### Eye Tracking Summary

**Figure 4.**
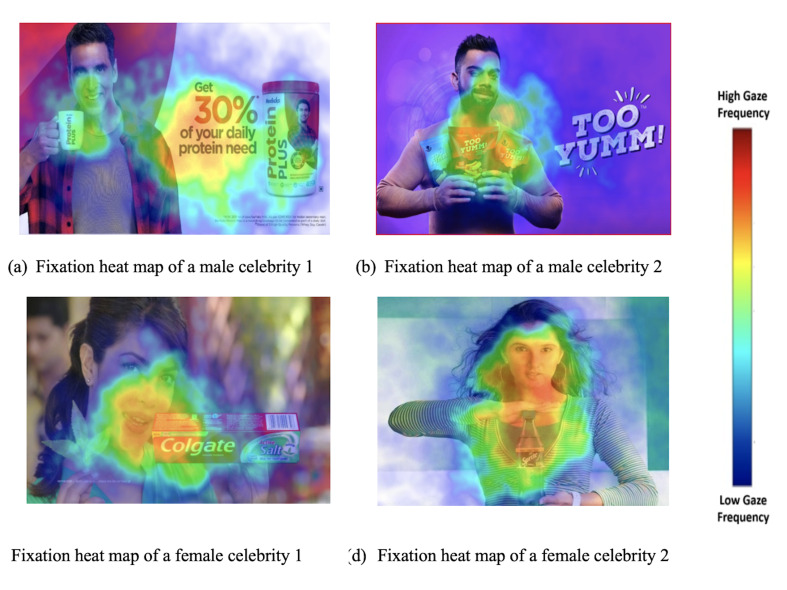
Visual attention heat maps of celebrity endorsers in the predefined AOI

Participants' viewing times and duration to the female and male celebrity spokespersons in the advertisements are compared using a Wilcoxon signed-rank test (two dependent samples, non-normally distributed data). The outcomes are displayed in Figure 4 and Table 3 below. According to the findings, participants' views count and dwell time of the female celebrity spokesperson in the advertisement was statistically higher than their male counter celebrity spokesperson.

Fixation count measures the number of fixations in a certain AOI, in the case of male celebrities it was found that mean and variance of fixation count was 10.930 and 37.716 whereas in the case of female celebrities it was 19.797 and 36.150 (Table 3). To the surprise dwell time mean and variance in case of male and female celebrities were far apart 3467.442 milliseconds and 5420.971milliseconds being the means and variance were 5388244.770 milliseconds and 4046503.819 milliseconds respectively.

It is clear from the Box plot and Scattergram charts that Fixation count and dwell time of female celebrity collectively is more than the male one. So, the hypothesis 1 is accepted. Consumers pay greater attention to female celebrities than male Celebrities.

The color coding of the heat map on figure 4 signifies the intensity of the attention on the stimuli. Red being the most intense, followed by yellow, green, and violet being the least intensity of the attention. It is evident from Figure 4, that males have lesser intense colors that females, which signifies that female celebrity get more attention their male counterpart. The p value has been computed by 1000 Monte Carlo Simulations with 99% confidence interval at alpha 0.05.

### Self- Reported Measure

i) *Purchase Intention*

According to self-reported statistics (Table 4), mean of purchase intention of male and female celebrities were 3.305 and 3.419 which were approximately close to each other. Similar was the case with the variance, standard deviation, and standard error of mean (Table 4).

The Wilcoxon test (two independent samples; non-normally distributed data; ordinal scale) is used to evaluate the differences in purchase intentions when the spokesperson is a female celebrity and male celebrity (see figure 6). The measuring scales' reliability is indicated by Cronbach's alpha values above 0.7 and for Purchase Intention it is 0.952. However, there is no significant difference in the degree of buy intents between a female celebrity-endorsed brand and a male celebrity spokesperson-endorsed brand (Table 7). As a result, hypothesis 2 was dismissed (Table 8).

**Table 3:**
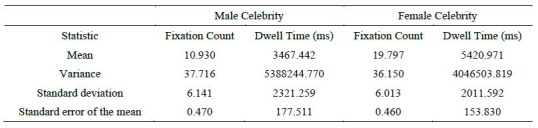
Eye tracking Measure Summary

**Figure 5.**
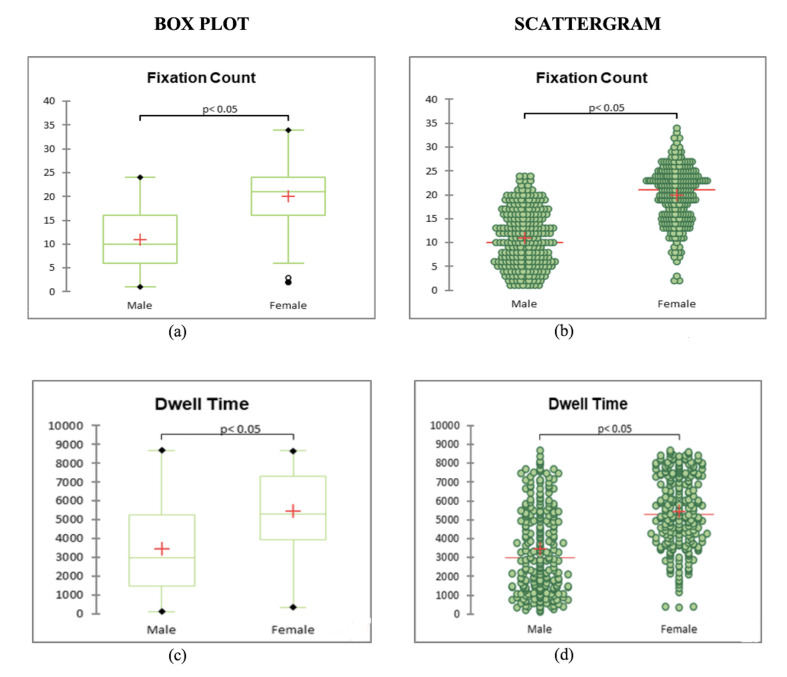
a) Box plot of fixation counts of male celebrity endorser on the celebrity area of interest; b) Scattergram of fixation count of Male Celebrity Endorser on the celebrity area of interest; c) Box plot of dwell time in milliseconds of male celebrity endorser on the celebrity area of interest d) Scattergram of dwell time of Male Celebrity Endorser on the celebrity area of interest

ii) *Celebrity Credibility*

The reliability of Celebrity credibility is measured by Cronbach Alpha which is coming out to be 0.947 which should be greater than 0.7. Hence the questionnaire is reliable. As mentioned earlier celebrity credibility is measured by three variables, namely perceived trustworthiness, expertise, and attractiveness, following are the results of the questionnaire,

a) Perceived Trustworthiness

Table 4 gives the statistics of the perceived trustworthiness in male as well as female celebrity endorser. Proceeding with the analysis, Figure 7a and 7b demonstrate that there is a substantial difference in the credibility of the male and female celebrity endorsers according to the Wilcoxon sign test (Table 7).

Furthermore, it is evident from the box plot and scattergram that male celebrities are more trustworthy than female celebrities, which accepts the 3a hypothesis—which holds that male celebrities advocate products that are more trustworthy (Table 8).

**Table 4:**
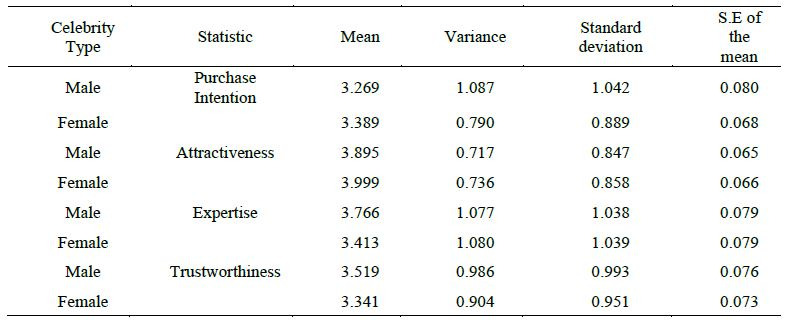
Self-Reported Data Statistics

**Figure 6.**
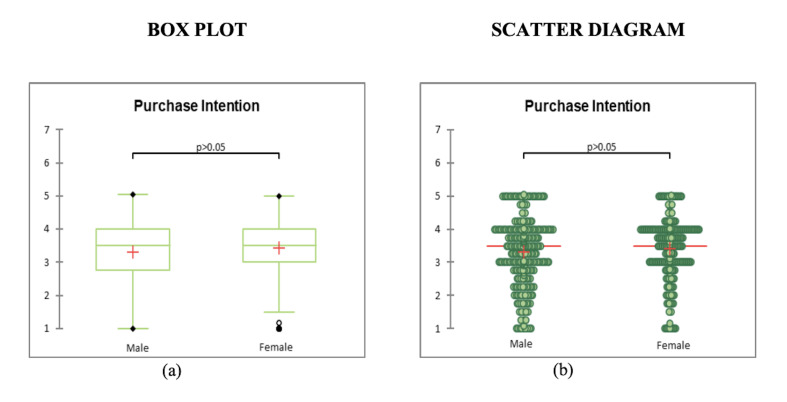
(a) Box plot of Purchase Intention Male Celebrity Endorser; (b) Scattergram of Purchase Intention of Male Celebrity Endorser

b) Perceived Attractiveness

Coming onto attractiveness, statistical details are given Table 4. From the Table 4 it is found out that attractiveness mean was close enough in the case of male and female celebrity (Male: Mean (3.895), Variance (0.717)) and (Female: Mean (3.999), Variance (0.736)). And as shown in Table 7 Wilcoxon test states that there is no significant difference between the observed attractiveness of a female and male celebrity endorser. Hence the 3b hypothesis is rejected (Table 8).

c) Perceived Expertise

**Figure 7.**
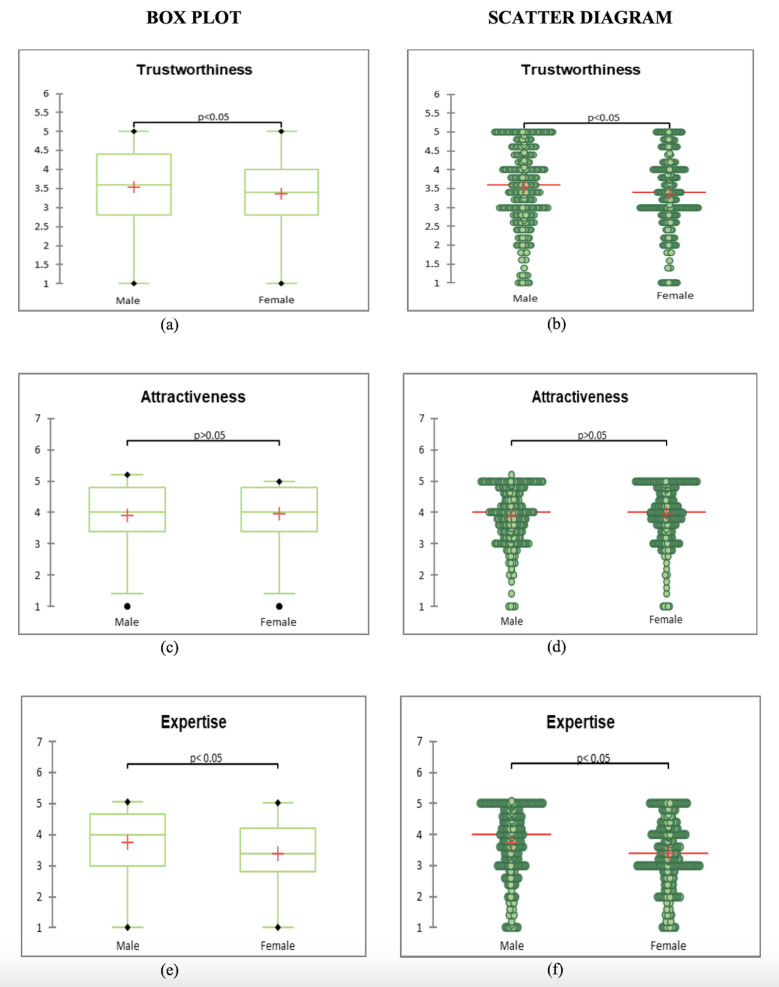
(a) Box plot of Perceived Trustworthiness of Male Celebrity Endorser; (b) Scattergram of Perceived Trustworthiness of Male Celebrity Endorser; (c) Box plot of Perceived Attractiveness of Male Celebrity Endorser; (d) Scattergram of Perceived Attractiveness of Male Celebrity Endorser; (e) Box plot of Perceived Expertise of Male Celebrity Endorser; (f) Scattergram of Perceived Expertise of Male Celebrity Endorser

After discussing trustworthiness and attractiveness, expertise was compared. The statistical analysis is shown in table 3 which shows a difference in the mean and variance of the male and female celebrity. It is found that there is a significant difference between expertise of a female and a male celebrity endorser as shown in the Figure 7e and 7f. And from box plot and scattergram of the same, it is evident that expertise of the male endorser is more than that of female endorser. Hence hypothesis 3c is rejected.

**Table 5:**
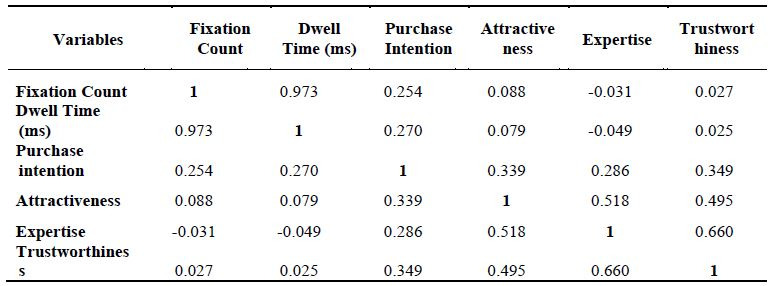
Correlation matrix: Attention of Male Celebrity Endorser and Celebrity Credibility

(ms: Milliseconds)

**Table 6:**
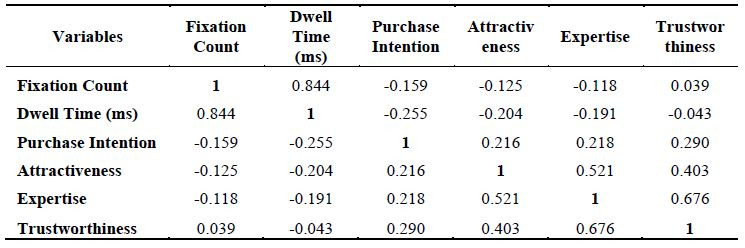
Correlation matrix: Attention of Female Celebrity Endorser and Celebrity Credibility

**Table 7:**
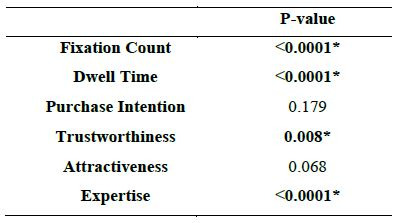
P-value of Wilcoxon signed-rank test / Two-tailed test

### Neuromarketing and Self-Reported measure

After studying, the correlation between the attention and purchase intention, it was found that attention is positively correlated to purchase intention in the case of male celebrity endorsement which means higher the attention higher the purchase intention (Table 5).

Hence, hypothesis 4a is accepted. But opposite is true in the case of female celebrity endorsement, thereby meaning that increasing the attention would result in lower purchase intention (Table 6) proving that hypothesis 4b is rejected. Also, it is having been found that in the case correlation between attention and celebrity credibility it is found that attention is positively correlated to male endorsers (Table 5 and the opposite is true in case of female endorser (Table 6).

**Table 8:**
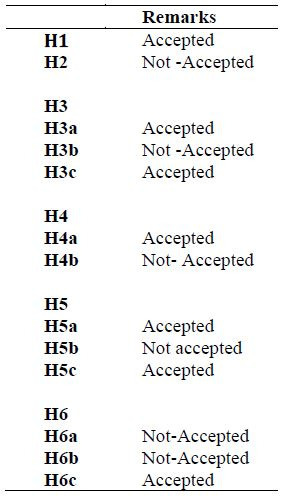
Hypothesis Testing Report

*p-value<0.05 is significant

The previous statement signifies that higher the attention, higher the male celebrity credibility but lower female celebrity credibility. By this it is clear that hypothesis 5 is accepted and 6 is rejected (Table 8).The next part goes into additional detail about the research findings, including a discussion of the theoretical and management implications.

## Discussion

In the Indian scenario of FMCG advertising, celebrity endorsement has the biggest influence. This research tries to understand the difference between celebrity gender casting in advertisements. The study attempts to find out the difference between male and female celebrity endorser using the combination of modern and traditional marketing method. By modern marketing, it is subjected to neuromarketing and traditional marketing research method which is used here is questionnaire. It has been stated by the authors that combination of neuroscientific methods and traditional methods give the best results. That is why this study has incorporated both methods to enhance the analysis.

The findings of the study suggest that female endorser has significantly more attention than male endorser in the FMCG advertising. The results from the study states that female celebrity endorser has more attention than male while viewing the advertisement is supported, stating that a greater number of fixations as well as dwell time. Literature suggests that longer the dwell time, longer is the cognition (15). Previous work suggest that male endorsers are dominant in the advertising industry as compared to the female endorser (28; 20).

Now that, this study revealed the females have greater attention than male which means greater cognition on the advertisement due to the presence of female celebrity endorser. This finding of the study does not align with previous work. When the attention is greater on the female endorser than male, it might imply that it will have a greater purchase intention. But this is not true, it has been found out that attention is negatively correlated to purchase intention in the case of female endorser. Greater the attention on the female endorser lesser is the purchase intention. Interestingly in the case of male celebrity, greater the attention greater is the purchase intention.

Also, the findings of the study reveal that there is no significant difference on the effect on the purchase intention between male and female celebrity endorsers. The findings do not align with the finding of Zhang and Yuan which states that eye movement is positively correlated to effectiveness of the advertisement. Celebrity credibility is one of the major factors leading to purchase intention. Starting with attraction element of the celebrity credibility, it was found that there was no significance difference between the attraction of female and male endorser even if it has more attention than the male one. 

However, perceived trustworthiness factor was in the favor of male celebrity. Male celebrities were trusted more which aligns with the past research. Male celebrities are considered to be trustworthy than female ones have been pointed out by several authors. Perceived expertise part of the celebrity credibility was expected to have more on the female celebrity which the third part of the third hypothesis which states that female celebrity is considered to have more opinion and expertise on the product and the advertisement compared to male celebrity. The result which comes out to be akin to the previous research, as male endorsers was considered to have expertise more than the female celebrity endorser (62). The study suggests that consumers rely more on the male endorser compared to the female endorser because of dominance of male endorsers and stereotyping of the genders in the advertisement (1). In the same context the attention is positively correlated to celebrity credibility in the case of male celebrity, but it is otherwise in the case of female celebrity. But when the consumers look at the female celebrity longer, credibility is decreased.

Lastly, the conclusion is that, although female celebrity endorser has more attention, but it does not guarantee that consumers would have purchase intention to buy the product just because of the presence of female celebrity. The credibility factor comes into play where attractiveness between male and female endorsers doses do not have any major difference. But the perceived trustworthiness and expertise have a role and is found to be more in the case of male celebrity. To let consumers favor for a product reliability is important and that’s what male endorsers do. They create a sense of trust and expertise which builds an opinion of sincerity which would let consumers think that buying products from the male celebrity endorsed products won’t disappoint them rather delight them. Trust and reliability are beyond attractiveness in the minds of the consumers. The attention on the product endorsed by female endorser does not guarantee the purchase intention as attention is negatively correlated to the purchase intention. But it is opposite in the case of male endorsed product. After discussing the study, we move one to the next and a very important section that states the managerial implications of the study.

### Limitations

This study has focused only on print advertisement in the digital form, future research can consider audio visual advertisements. Since it is a laboratory setting experiment and participants were aware that they were being tracked, so the results could have been manipulated (89) but we are bound to make participants aware about the eye-tracking because of the ethical norms. The other limitation is that we have compared male and female celebrities individually and not considering the couple advertisement. A comparison of male versus couple and similarly female versus couple celebrity endorsement could be studied. In this study an issue which has been raised is the low involvement products like FMCG, scholars can study about high involvement products like automobile, gold, high end appliances or real estate. The study is also limited to products; celebrity endorsement in services could also be studied for better understanding of the Indian Celebrity Endorsement scenario.

The future research will consider the following points to strengthen conclusions regarding the impact of celebrity endorsement and gender on purchase intentions: Firstly, future studies will include control conditions with advertisements featuring only the product, without any celebrities. This will provide a clearer baseline for comparison, helping to isolate the effect of celebrity endorsement on purchase intentions. Secondly, a comparative analysis between advertisements with and without celebrities will be conducted to offer insights into the specific impact of the celebrity's presence. Measuring the differences in purchase intentions across these conditions will elucidate the role of celebrity endorsements. Moreover, beyond demographic factors such as age, future research will consider variables like participants' interests, past experiences, attitudes toward celebrities and advertised products, as well as individual skills and knowledge (e.g., memory span). These factors can influence the process of examining Areas of Interest (AOI) zones and evaluating advertisements. Finally, all results will be analyzed separately for male and female participants to provide a more detailed understanding of gender-specific responses to celebrity endorsements.

### Ethics and Conflict of Interest

The author(s) declare(s) that the contents of the article are in agreement with the ethics described in http://biblio.unibe.ch/portale/elibrary/BOP/jemr/ethics.html and that there is no conflict of interest regarding the publication of this paper. The ethical approval was given by Institute Ethics Committee, Indian Institute of Information Technology, Allahabad, India.
